# RAM/Fam103a1 Is Required for mRNA Cap Methylation

**DOI:** 10.1016/j.molcel.2011.08.041

**Published:** 2011-11-18

**Authors:** Thomas Gonatopoulos-Pournatzis, Sianadh Dunn, Rebecca Bounds, Victoria H. Cowling

**Affiliations:** 1 Division of Cell Signalling and Immunology, College of Life Sciences, University of Dundee, Dow Street, Dundee DD1 5EH, UK

## Abstract

The 7-methylguanosine cap added to the 5′ end of mRNA is required for efficient gene expression in eukaryotes. In mammals, methylation of the guanosine cap is catalyzed by RNMT (RNA guanine-7 methyltransferase), an enzyme previously thought to function as a monomer. We have identified an obligate component of the mammalian cap methyltransferase, RAM (RNMT-Activating Mini protein)/Fam103a1, a previously uncharacterized protein. RAM consists of an N-terminal RNMT-activating domain and a C-terminal RNA-binding domain. As monomers RNMT and RAM have a relatively weak affinity for RNA; however, together their RNA affinity is significantly increased. RAM is required for efficient cap methylation in vitro and in vivo, and is indirectly required to maintain mRNA expression levels, for mRNA translation and for cell viability. Our findings demonstrate that RAM is an essential component of the core gene expression machinery.

## Graphical abstract

**Graphical Abstract:**

**Figure:**
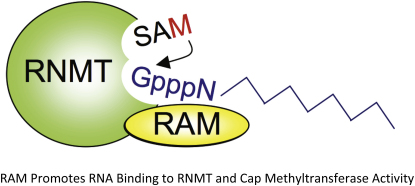

**Highlights:**

► RAM/Fam103a1 is a component of the human cap methyltransferase complex ► RAM is required for efficient cap methylation in vitro and in vivo ► RAM activates RNMT (cap methyltransferase), and increases RNMT affinity for RNA ► RAM is indirectly required to maintain mRNA levels and for mRNA translation

## Introduction

The methyl cap is the inverted 7-methylguanosine group linked to the first transcribed nucleotide of RNA polymerase (pol) II transcripts in eukaryotes (Cowling, 2009; Shatkin, 1976; Shuman, 2002). The 7-methylguanosine is linked by the 5′ hydroxyl group through a triphosphate linkage to the transcript, to create a structure designated m7G(5′)ppp(5′)X (X is the first transcribed nucleotide). This is a unique molecular structure within the cell and is thought to specifically target the 5′ end of RNA pol II transcripts for several gene regulatory processes, including splicing, nuclear export of mRNA, and translation initiation (Bentley, 2005; Cowling, 2009; Moore and Proudfoot, 2009). The methyl cap also protects RNA from exonucleases until it is removed by decapping enzymes (Liu and Kiledjian, 2006).

The enzymes which catalyze methyl cap synthesis are essential from yeast to man; in mammals these are RNGTT (RNA guanylyltransferase and 5′ triphosphatase) and RNMT (RNA guanine-7 methyltransferase) (Chu and Shatkin, 2008; Furuichi and Shatkin, 2000; Shuman, 2002). Nascent RNA is transcribed with a 5′ triphosphate on the first transcribed nucleotide. RNGTT has two active sites that catalyze removal of the terminal phosphate and addition of guanosine monophosphate to create the inverted guanosine cap, G(5′)ppp(5′)X. RNMT catalyzes methylation of the cap at the N-7 position to create the methyl cap, m7G(5′)ppp(5′)X. RNMT can only catalyze methylation of guanosine when it is a component of a cap structure attached to a transcript. Inhibition of RNMT expression results in loss of cap methylation and cell viability, and therefore, if there is another as yet undiscovered cap methyltransferase, it is not fully redundant with RNMT (Chu and Shatkin, 2008; Cowling, 2010; Shafer et al., 2005).

The majority of experimental data indicates that synthesis of the methyl cap occurs predominantly during the early stages of transcription. In a similar manner to other pre-mRNA processing events, the mechanics of methyl cap formation are structured on the RNA pol II C-terminal domain (CTD) (Bentley, 2005; Chapman et al., 2008; Moore and Proudfoot, 2009). RNGTT and RNMT are recruited to the TFIIH-phosphorylated CTD, increasing the local concentration of these enzymes in the environment of the emergent nascent transcript.

Methylation of the guanosine cap has recently been demonstrated to be regulated in yeast and mammalian cells. In mammals, c-Myc and E2F-1 were found to upregulate cap methylation on a subset of their transcriptional targets and other transcripts (Cole and Cowling, 2009; Cowling and Cole, 2007; Fernandez-Sanchez et al., 2009). c-Myc-induced cap methylation was correlated with c-Myc-induced protein synthesis and cell proliferation, and inhibition of the cap methylation reaction was synthetic lethal with elevated c-Myc expression (Fernandez-Sanchez et al., 2009). In addition, cap methylation was demonstrated to be enhanced by Importin-α, which increases RNMT activity (Wen and Shatkin, 2000). In yeast, methyl cap levels were found to be regulated in response to glucose and amino acid deprivation, which may be a mechanism to temper translation while metabolic resources are limiting (Jiao et al., 2010).

Although formation of the methyl cap is a critical step in gene expression, the mechanistic details of this process are limited. We purified human cap methyltransferase complexes and identified a previously uncharacterized protein, RAM/Fam103a1, which is required for cap methylation in vitro and in vivo and indirectly required to maintain mRNA levels, for mRNA translation and cell viability.

## Results

### Fam103a1/RAM Is a Component of the Human Cap Methyltransferase Complex

**Figure 1:**
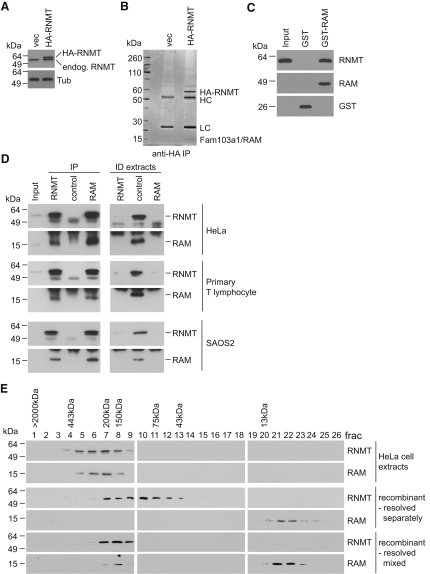
Fam103a1/RAM Isolated in Human RNMT Complexes (A) RNMT was detected by western blot in extracts from 293 cell lines expressing HA-RNMT and vector control. β-Tubulin (Tub) was detected as a loading control. (B) HA-RNMT complexes were purified using anti-HA antibodies, resolved by SDS-PAGE and stained with Coomassie Blue. Migration of HA-RNMT, antibody heavy chain (HC), light chain (LC) and Fam103a1/RAM are indicated. (C) Recombinant RNMT (Input) was mixed with GST and GST-RAM, and affinity purified on glutathione agarose. Proteins eluted were analyzed by western blot to detect RNMT, RAM, and GST. (D) Immunoprecipitations were performed on HeLa cell, primary T lymphocyte, and SAOS-2 cell extracts, using the antibodies indicated. Western blots were performed to detect RAM and RNMT in inputs, immunoprecipitates (IP), and immunodepleted extracts (ID extracts). (E) Gel filtration on a Superdex s200 10/30 column was used to resolve 1 mg HeLa cell extract, 1 μg recombinant RNMT, 1 μg RAM, and 1 μg of a 1:1 mixture of RNMT and RAM; 0.5ml fractions were collected following the void volume. Western blots were performed to detect RAM and RNMT. The migration of standards is indicated. (See also Figure S1.)

In order to identify components of the human cap methyltransferase, HA (hemagglutinin)-tagged RNMT expressed in human embryonic kidney 293 cells (Figure 1A), was immunoprecipitated from cell extracts, resolved by SDS-PAGE and proteins stained with Coomassie Blue (Figure 1B). A 14 kDa protein that copurified with RNMT was identified by mass spectrometry as Fam103a1, a protein of unknown function. We designated Fam103a1 as RAM (RNMT-Activating Mini-protein). Recombinant RNMT was found to bind directly to recombinant GST (glutathione S-transferase)-tagged RAM but not to GST alone (Figure 1C).

The interaction between endogenous cellular RNMT and RAM was confirmed by immunoprecipitations performed on extracts of HeLa cells (human cervical adenocarcinoma-derived), human primary T lymphocytes, and SAOS-2 cells (human osteosarcoma-derived) (Figure 1D, left panels). By western blotting, RAM was detected in RNMT immunoprecipitates and RNMT was detected in RAM immunoprecipitates. In order to determine what proportion of cellular RAM and RNMT are present in the same complex, antibodies raised against RAM or RNMT were used to efficiently immunodeplete their target from cell extracts, as determined by western blot (Figure 1D, right panels). RAM was also efficiently cleared from cell extracts depleted of RNMT, and RNMT was efficiently cleared from extracts depleted of RAM, indicating that most cellular RNMT and RAM are present in RNMT-RAM complexes.

Gel filtration analysis of HeLa cell extracts and recombinant proteins was performed to determine what proportion of cellular RAM and RNMT are present in complexes and as monomers (Figure 1E). Recombinant RNMT and RAM were resolved by gel filtration in order to observe the migration of the monomeric proteins (peak fractions 10 and 21/22, respectively, Figure 1E, middle panels). Recombinant RAM and RNMT were also mixed prior to resolution by gel filtration, which resulted in peak elution of both proteins in the same fraction (fraction 8), indicating that they are forming a higher molecular weight complex (Figure 1E, lower panels). Cellular RNMT and RAM resolved in gel filtration in approximately 200 kDa complexes, (peak fractions 6/7, Figure 1E, upper panels), that is, they were not detected as monomers and migrated in larger complexes than the recombinant RAM-RNMT complexes. This indicates that a relatively large proportion of cellular RAM-RNMT complexes contain one or more additional proteins compared to the recombinant complex. Our proteomic analyses have indicated that several other proteins interact with the RNMT-RAM complex, although all at substoichiometric ratios (data not shown). It is likely that only one RNMT and one RAM protein is present in each RNMT-RAM complex, since RNMT expressed with two different tags could not be coimmunoprecipitated, and the same was true for RAM (data not shown).

Recombinant RNMT had been found to interact with RAM in the absence of RNA (Figure 1C), and RNase treatment did not influence the RNMT-RAM coimmunoprecipitation performed on HeLa cell extracts (Figure S1), indicating that RNA is not required to mediate their interaction.

### *H.sapiens* RAM Is a 118 Amino Acid Nuclear Protein

**Figure 2:**
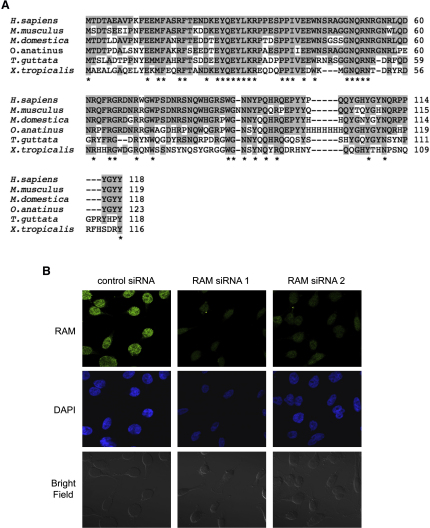
RAM Is Conserved in Vertebrates (A) The amino acid sequence of the *H.sapiens* RAM protein (NP_113640.1) and homologs in *M. musculus* (NP_080273.1), *M. domestica* (XP_001362351.1), *O. anatinus* (XP_001513424), *T. guttata* (XP_002199043.1), and *X. tropicalis* (NP_001037960.1) were aligned using EMBL-EBI ClustalW2 Multiple Sequences Alignment software, using the default parameters (Chenna et al., 2003). Amino acids identical in *H.sapiens* RAM protein and at least one other species are highlighted in gray, and those identical in all species investigated are indicated (^∗^). (B) Immunofluorescence microscopy was used to detect RAM expression in HeLa cells. Cells were transfected with two independent siRNAs directed against RAM and a nontargeting control for 48 hr prior to fixation to confirm specificity of RAM staining. DAPI stain was used to detect nuclei.

*H.sapiens* RAM is a 118 amino acid protein (Figure 2A). RAM homologs were identified in vertebrates, and an alignment of RAM proteins from a spectrum of vertebrate species is depicted in Figure 2A. The N-terminal region is well conserved with 25 of the N-terminal 55 amino acids being identical in all species examined. Amino acids 56–90 are enriched in asparagine and arginine residues (NR-rich) and the C-terminal amino acids 91–118 are enriched in proline, glutamine, and tyrosine residues (QYP-rich). Functional domains previously established in other proteins were not identified in RAM. However, the RAM C terminus has a similar enrichment of amino acids to the C-terminal, RNA-binding domain of the hnRNPU protein family (Kiledjian and Dreyfuss, 1992), which raised the possibility that RAM is a RNA-binding protein.

RNMT is a nuclear protein (Wen and Shatkin, 2000) (Figure 6A), and as expected, RAM was also observed to have a predominantly nuclear localization by immunofluorescence (Figure 2B). siRNA directed against RAM was used to demonstrate the specificity of our anti-RAM antibodies.

### RAM Enhances RNA Recruitment to RNMT

**Figure 3:**
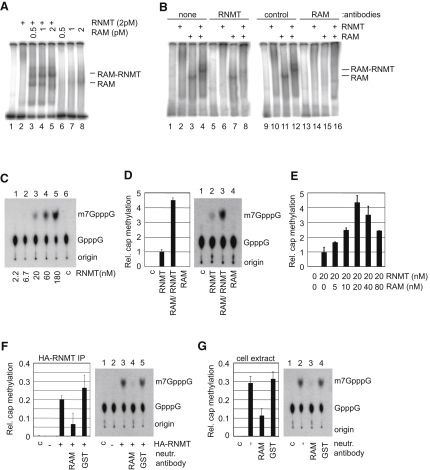
RAM Is a RNA-Binding Protein and Promotes Cap Methylation (A) RAM and RNMT interaction with RNA was investigated by RNA band shift assay. 2 pmol RNMT and/or 0.5–2 pmol RAM were incubated with an excess of ^32^P-capped transcript, and complexes were resolved by gel electrophoresis. (B) As in (A) except prior to gel electrophoresis, 2 pmol of RNMT and/or 2 pmol RAM were incubated with 700 ng anti-RNMT, GST, or RAM antibodies. The position of RAM and RNMT-RAM complexes is indicated. Assays were repeated three times, and a representative result is shown. (C) Cap methyltransferase assay was performed using a titration of recombinant RNMT, molarity indicated, or no protein was added (C). RNMT was incubated with ^32^P-capped transcript and s-adenosyl methionine. Following the reaction, GpppG and m7GpppG were resolved by thin layer chromatography, as indicated. (D) The cap methyltransferase assay was performed with 20 nM RNMT and/or 20 nM RAM. (E) As in (D), except a titration of RAM was used. (F) Cap methyltransferase assay was performed on cellular HA-RNMT immunoprecipitated via the HA tag (lanes 3–5), or the same immunoprecipitation was performed on control cell extracts (lane 2). Prior to the assay, immunoprecipitates were incubated with anti-GST (lane 4) or anti-RAM antibodies (lane 5). (G) Cap methyltransferase assay was performed on 1 μg 293 cell extract (lanes 2–4). Prior to the assay, extracts were incubated with anti-GST (lane 3), or anti-RAM antibodies (lane 4). For Figures 3D–3G, mean relative cap methylation for four independent experiments and standard deviation is depicted (left panels). (See also Figure S2.)

The hypothesis that RAM binds to RNA was tested using a RNA band shift assay (Wen and Shatkin, 2000). A ^32^P-labeled transcript was incubated with recombinant proteins and the resultant protein-RNA complexes were visualized by retarded RNA migration on native PAGE. When 2 pmol RAM was incubated with RNA, an RNA-RAM complex was detected (Figure 3A, lane 8). The presence of RAM in the complex was confirmed by incubating the mixture of RNA and RAM with anti-RAM antibodies prior to resolution on PAGE, which inhibited complex formation (Figure 3B, compare lanes 3 and 15), whereas anti-RNMT and anti-GST antibodies had no effect (lanes 7 and 11).

When 2 pmol RNMT was incubated with RNA, a complex was undetectable (Figure 3A, lane 2). (However, higher concentrations of RNMT result in the observation of a RNMT-RNA complex [Wen and Shatkin, 2000]). Incubation of RAM with RNMT produced a RNA complex which migrated more slowly than the RNA-RAM complex (Figure 3A, lanes 3–5). The presence of RAM and RNMT in this complex was confirmed by its neutralization with anti-RAM and RNMT antibodies (Figure 3B, compare lane 4 with lanes 8 and 16), but not anti-GST antibodies (lane 12). As described above, when RAM alone was incubated with RNA, the RNA-RAM complex was first observed with 2 pmol RAM (Figure 3A lane 8), however, the RNA-RAM-RNMT complex was visible with 0.5 pmol RAM in the presence of 2 pmol RNMT (Figure 3A, lane 3), indicating that RNA cooperatively binds to RAM and RNMT.

A previous publication has demonstrated that RNMT binds efficiently to capped (GpppX), but not uncapped (pppX) or methylcapped (m7GpppX) transcripts, presumably because the cap interacts with the active site (Fabrega et al., 2004; Wen and Shatkin, 2000). RAM complexes bound equivalently to uncapped, capped, and methylcapped transcripts (Figure S2A), consistent with RAM binding to RNA rather than the cap structure.

### RAM Increases RNMT Cap Methyltransferase Activity

Since RAM forms a complex with RNMT and RNA, the hypothesis that RAM regulates RNMT cap methyltransferase activity was investigated using an in vitro cap methyltransferase assay (Pillutla et al., 1998). A ^32^P-labeled, capped transcript was incubated with recombinant proteins and the methyl donor s-adenosyl methionine. Methylation of the cap was detected by thin layer chromatography and quantitated by phosphoimager. Recombinant RNMT was observed to catalyze methylation of the cap in a dose-dependent manner (Figure 3C). Incubation of RNMT with an equimolar concentration of recombinant RAM increased cap methylation by over 4-fold (Figure 3D, lanes 2 and 3). RAM did not exhibit cap methyltransferase activity independently of RNMT (Figure 3D, lane 4). Titrating the concentration of RAM used in the reaction increased cap methylation in a dose-dependent manner, with an equimolar concentration of RAM and RNMT resulting in the highest cap methyltransferase activity (Figure 3E). When the molar concentration of RAM was higher than that of RNMT, cap methylation decreased. One possible explanation is that excess RAM titrates RNA away from the cap methyltransferase complex.

In order to investigate whether RAM was required for cellular RNMT activity, the cap methyltransferase assay was performed using HA-RNMT immunoprecipitated from 293 cell extracts (Figure 3F). Immunoprecipitated RNMT exhibited cap methyltransferase activity, and this was significantly reduced by including anti-RAM but not anti-GST antibodies in the reaction (Figure 3F, lanes 4 and 5). In order to determine whether total cellular cap methyltransferase activity is dependent on RAM, the cap methyltransferase assay was performed on untreated HeLa cell extracts (Figure 3G). Cap methyltransferase activity was exhibited by cell extracts, and this was significantly reduced by including anti-RAM but not anti-GST antibodies in the reaction (Figure 3G, lanes 3 and 4). Cap methyltransferase activity was also unaltered by incubating the reaction in anti-HA, anti-eIF4A1, and anti-PABP antibodies (Figure S2B).

### The RAM N Terminus Interacts with RNMT Methyltransferase Domain

**Figure 4:**
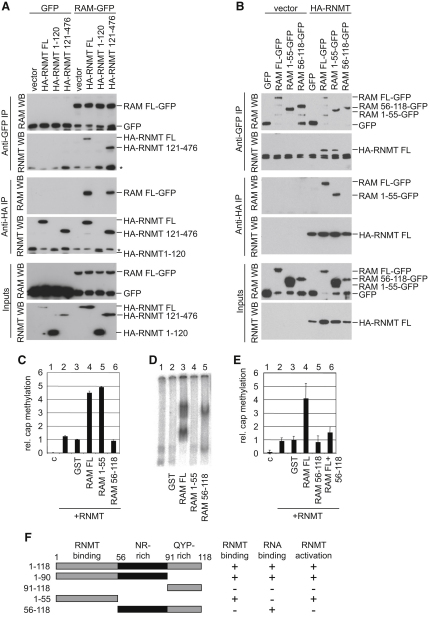
RAM N Terminus Activates RNMT (A and B) 293 cells were transfected with combinations of pEGFP-RAM and pCDNA-HA-RNMT, deletion mutants, and vector controls. Immunoprecipitations were performed with anti-HA and anti-GFP antibodies. Western blots were performed to detect RAM and RNMT in inputs, anti-HA antibody immunoprecipitates, and anti-GFP antibody immunoprecipitates. (^∗^) Indicates a cross-reacting band. (C) Cap methyltransferase assay was performed as in Figure 3D using 20 nM RNMT plus 20 nM GST or GST-RAM protein. (D) RNA band shift assay was performed as in Figure 3A using 2 pmol GST or GST-RAM protein. (E) Cap methyltransferase assay was performed as in (C) except for lane 6, in which RNMT was incubated with GST-RAM FL and 56–118 (all 20 nM). (F) Summary of RAM domain analysis. Deletion mutants used and their activity in RNMT binding, RNA binding, and RNMT activation are depicted. For Figures 4C and 4E, mean relative cap methylation for four independent experiments and standard deviation is depicted. (See also Figure S3).

In order to further probe the mechanism of RAM function, the domains of RAM and RNMT which interact were investigated. In 293 cells, RAM was expressed fused to Green Fluorescent Protein on the C terminus (RAM-GFP), and RNMT was fused to the HA epitope on the N terminus (HA-RNMT). RAM-GFP and HA-RNMT, and deletion mutants thereof, were immunoprecipitated via their GFP and HA tags respectively. HA-RNMT was coimmunoprecipitated with RAM-GFP but not GFP alone (Figures 4A and 4B, upper panels). Conversely, RAM-GFP but not GFP alone was coimmunoprecipitated with HA-RNMT (Figures 4A and 4B, middle panels). This confirms the interaction of RAM and RNMT observed in Figure 1 using an alternative set of antibodies.

In order to identify the interacting regions of RNMT and RAM, mammalian expression vectors were designed to express deletion mutants of these proteins. These were RNMT amino acids 1–120 and 121–479 and RAM amino acids 1–55, 56–118, 1–90, and 91–118. RNMT 1–120 is not required for RNMT cap methyltransferase activity but contains two nuclear localization signals (NLSs), whereas RNMT 121–479 contains the cap methyltransferase domain and an additional NLS (Saha et al., 1999; Shafer et al., 2005). RAM mutants were designed based on the nature of their constituent amino acids, as discussed earlier (Figures 2A and 4F).

RAM-GFP and GFP were coexpressed with HA fusions of full-length RNMT (FL), RNMT 1–120, RNMT 121–476 and vector control. RAM was observed to interact with RNMT FL and RNMT 121–476 but not RNMT 1–120 (Figure 4A). HA-RNMT and vector control were coexpressed with GFP fusions of RAM FL, RAM 1–55, RAM 56–118, and GFP alone. HA-RNMT was observed to interact with RAM FL and RAM 1–55 but not RAM 56–118 (Figure 4B). HA-RNMT was also observed to interact with RAM 1–90 but not RAM 91–118 (Figure S3).

### The RAM N Terminus Activates RNMT

The cap methyltransferase assay was performed to determine the effect of the RAM deletion mutants on RNMT cap methyltransferase activity (Figure 4C). RAM 1–55 activated RNMT-dependent cap methylation equivalently to wild-type RAM, whereas RAM 56–118 did not. RAM 56–118 did not inhibit the basal activity of RNMT, indicating that although it binds to RNA (Figure 4D), at the concentration used in the assay it did not prevent the interaction of RNMT and RNA. RAM 1–90 also activated cap methylation whereas RAM 91–118 did not (Figure S3B). Therefore only the N-terminal mutants of RAM that were observed to interact with RNMT activated cap methylation (summarized in Figure 4F).

The RNA band shift assay was performed with the RAM deletion mutants in order to determine which domain binds to RNA. RAM 56–118 exhibited RNA-binding activity whereas RAM 1–55 did not (Figure 4D). RAM 1–90 exhibited RNA binding whereas RAM 91–118 did not (Figure S3C). These findings are summarized in Figure 4F.

In order to investigate whether the RNA-binding domain is utilized by RAM during cap methylation, the in vitro cap methyltransferase assay was performed with RNMT and RAM FL, in the presence or absence of RAM 56–118 (the RNA-binding domain) (Figure 4E). As had been observed previously, RAM FL stimulated RNMT activity (Figure 4E, lane 4), however, including RAM 56–118 in the assay with RAM FL significantly reduced this stimulatory effect (Figure 4E, lane 6). This suggests that RAM 56–118 titrates RNA away from RAM FL, and that RAM FL binds to RNA during the cap methylation reaction. The implications of this finding for cellular cap methylation are explored in the discussion.

### RAM Is Required for RNMT Expression and Cap Methyltransferase Activity In Vivo

**Figure 5:**
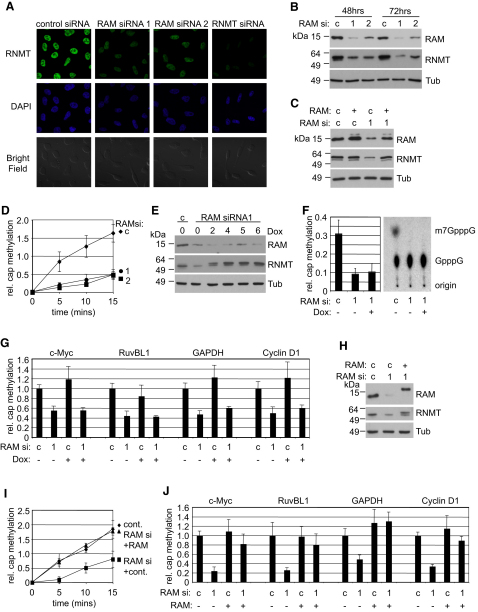
RAM Is Required for Cellular RNMT Expression and Cap Methylation Expression of RAM was reduced in HeLa cells by transfection of two independent siRNAs (1 or 2) or control (c) siRNA for 48 hr. (A) Immunofluorescence microscopy was used to detect RNMT expression and DAPI stain was used to detect nuclei. (B) Western blots were performed to detect RAM, RNMT, and Tubulin. (C) Cells were transfected with pcDNA5-RAM (+) or pcDNA5 (c), 48 hr later they were transfected with RAM siRNA (1) or control (c), and 48 hr later they were lysed. Western blots were performed as in (B). (D) Relative cap methyltransferase activity was determined in cell extracts following siRNA transfection. (E) RAM expression was depleted by transfection of siRNA for 24 hr and RNMT expression was Doxycycline-induced (Dox) for the time course indicated. Western blots were performed as in (B). (F) Relative cap methyltransferase activity in cell extracts was determined following 24 hr RAM siRNA transfection and 2 hr RNMT induction (Dox). (G) Methyl cap levels on the four endogenous transcripts indicated was determined relative to total transcript level following 24 hr RAM siRNA transfection and 6 hr RNMT induction (Dox). (H) Cells were transfected with RAM siRNA, 24 hr later they were transfected with 0.1 ug (+) pcDNA5 RAM and RNMT, and 24 hr later they were lysed. Western blots were performed as in (B). (I) Relative cap methyltransferase activity was detected in cell extracts. (J) Methyl cap levels on the four endogenous transcripts indicated was determined relative to total transcript level. In charts, average result and standard deviation of at least three independent experiments are depicted. (See also Figure S4).

In order to investigate whether RAM is required for cellular cap methylation, RAM expression was inhibited in HeLa cells using siRNA (Figure 5). Two independent siRNAs and siRNA-resistant cDNAs were used to control for siRNA “off-target” effects.

Prior to investigating cap methyltransferase activity, it was important to determine whether RAM was regulating RNMT expression or localization. RNMT is a nuclear protein, and its correct localization is essential for cell viability (Shafer et al., 2005; Wen and Shatkin, 2000). Inhibition of RAM expression did not result in a change in RNMT localization, as observed by immunofluorescence, but did result in a loss of RNMT expression (Figure 5A). This result was confirmed by western blot (Figure 5B). Conversely, inhibition of RNMT expression using siRNA resulted in loss of RAM expression (Figure S4). Expression of RAM from a RAM siRNA-resistant expression vector was sufficient to maintain RNMT protein expression while cells were transfected with RAM siRNA, confirming that loss of RNMT is not due to off-target effects of the RAM siRNA (Figure 5C).

As expected, inhibition of RAM expression (and concurrent loss of RNMT expression) resulted in a loss of cellular cap methyltransferase activity (Figure 5D). In order to determine whether RAM is required for RNMT activity in cells, RNMT expression was induced from a Doxycycline-regulated construct while RAM expression was inhibited by siRNA (Figure 5E). As observed previously, transfection of RAM siRNA repressed expression of RAM and RNMT. Following 2 hr of RNMT induction, RNMT levels were equivalent to those found in control cells, whereas RAM levels remained repressed for the time course shown (Figure 5E). Inhibition of RAM expression resulted in a loss of cap methyltransferase activity, however, restoration of endogenous levels of RNMT by a 2 hr Doxycycline induction (or longer), did not restore cap methyltransferase activity, confirming that RNMT requires RAM to function in vivo (Figure 5F). Inhibition of RAM expression also resulted in a reduction in the level of methyl caps on four endogenous transcripts investigated, c-Myc, RuvBL1, GAPDH, and Cyclin D1, as determined by anti-methyl cap immunoprecipitation and gene-specific RT-PCR (Figure 5G). Restoration of RNMT expression independently of RAM by 6 hr Doxycyline treatment did not restore methyl cap levels on endogenous transcripts (Figure 5G), and therefore RAM is required for RNMT function in vivo.

It was necessary to confirm that rescue of cap methyltransferase activity is possible following inhibition of RAM expression and that RAM siRNA is not simply causing irreparable damage to the cell. Therefore, 24 hr following RAM siRNA transfection, expression of RNMT and RAM was restored to endogenous levels by transfection of expression vectors (Figure 5H). The loss of cap methyltransferase activity (Figure 5I), and the loss of methyl cap levels on endogenous transcripts (Figure 5J), observed following RAM siRNA transfection were reversed by restoring RAM and RNMT expression to endogenous levels.

### RAM Expression Is Required for RNA Pol II Transcript Maintenance and Translation

**Figure 6:**
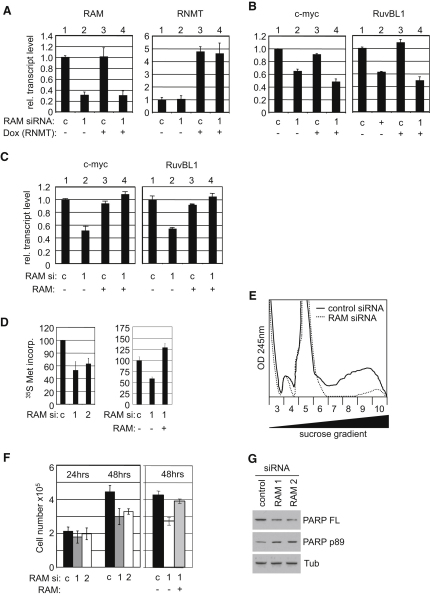
RAM Is Required for RNA Pol II Transcript Maintenance RAM siRNA (1) and controls (c) were transfected into HeLa cells for 24 hr and RNMT was induced with Doxycycline for 6 hr (+).RAM and RNMT transcripts (A) and c-Myc and RuvBL1 transcripts (B) were detected by real-time PCR. (C) 24 hr following RAM siRNA treatment 0.1 ug (+) pcDNA5 RAM and RNMT were transfected for 24 hr. RNA was harvested and the transcripts indicated were detected by real-time PCR. (D) Cells were prepared as in (C). Relative incorporation of ^35^S-cysteine and ^35^S-methionine into cellular proteins was determined. (E) Polysome profiles of cells transfected with RAM or control siRNA were determined. A representative result for five independent experiments is shown. (F) Cells were prepared as in (D). Cell counts were determined. (G) 48 hr following RAM siRNA, western blots were performed to detect full-length (FL) and cleaved (p89) PARP. In charts, average result and standard deviation of at least three independent experiments are depicted.

The methyl cap has been demonstrated to stabilize transcripts (Furuichi et al., 1977). Since RAM is required in vivo for the formation of the methyl cap on transcripts, then it was reasoned that it should also be required to maintain expression of these transcripts. As expected, following inhibition of RAM expression using siRNA (Figure 6A, lanes 1 and 2), RNA pol II transcript levels were found to be depleted, including c-Myc and RuvBL1 (Figure 6B, lanes 1 and 2), which correlated with a loss in the stability of these transcripts (data not shown). Clearly maintenance of all cellular transcripts was not dependent on the cap methyltransferase, since endogenous RNMT expression was not affected by RAM depletion (Figure 6A, lanes 1 and 2).

Since RAM is required for RNMT cap methyltransferase activity in vivo (Figure 5G), expression of RNMT without RAM should be insufficient to maintain RNA pol II transcript levels. In cells in which RAM expression had been inhibited by siRNA, RNMT expression was restored by activating a Doxycycline-regulated RNMT construct for 6 hr (Figures 5E and 6A, lanes 3 and 4). Expression of RNMT without RAM could not restore c-Myc or RuvBL1 transcript levels (Figure 6B, lane 4). In order to discount that RAM siRNA was not simply causing irreparable damage to the transcriptome, 24 hr following RAM siRNA transfection RAM and RNMT expression was restored as in Figure 5H. The RAM siRNA-dependent loss of c-Myc and RuvBL1 expression (Figure 6C, lane 2), could be reversed by restoring RAM and RNMT expression (Figure 6C, lane 4).

The methyl cap binds to eIF4E and is required for efficient translation initiation, under most circumstances (Gingras et al., 1999). For this reason, and since RAM is required for RNA pol II transcript maintenance (Figure 6B), loss of RAM was predicted to indirectly result in loss of protein synthesis. As expected, protein synthesis was found to be reduced in response to inhibition of RAM expression, as determined by incorporation of labeled amino acids into cellular proteins, and this effect could be reversed by restoring RAM and RNMT expression (Figure 6D). Cellular protein synthesis rates can also be inferred by “polysome profiling,” a technique in which free ribosomes can be seperated from mRNA bound ribosomes (polysomes) on a sucrose gradient (Figure 6E, lanes 7 to 10). Inhibition of RAM expression resulted in a loss of polysomes, consistent with loss of mRNA translation. Inhibition of RAM expression also resulted in a loss of cell accumulation (Figure 6F), including a mild induction of apoptosis, as determined by cleavage of the apoptotic marker PARP (Figure 6G).

### RAM Regulates RNMT Expression by a Posttranscriptional Mechanism

**Figure 7:**
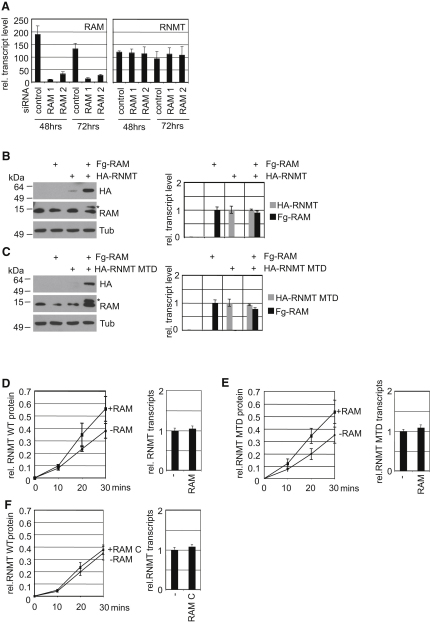
RAM Is Required for RNMT Translation and Stability (A) RAM and RNMT transcript level was determined by RT-PCR in HeLa cells transfected with two independent RAM-directed or control siRNAs, for 48 or 72 hr. (B) 293 cells were transfected with pcDNA3.1 Fg-RAM and HA-RNMT or relavent controls for 2 days. Western blots were performed to detect HA-RNMT (HA), RAM (^∗^ indicates Fg-RAM), and Tubulin. RT-PCR was performed to detect Fg-RAM and HA-RNMT transcripts. (C) as (B), except pcDNA3.1 HA-RNMT-MTD (methyltransferase dead) replaced HA-RNMT. (D) RNMT or RNMT and RAM were in vitro translated in the same reaction. At the times indicated, RNMT protein levels were quantitated. At 30 min RNMT and RAM transcripts were quantitated by RT-PCR. Values were normalized to input DNA and the average and standard deviation of three independent experiments are depicted. (E) as (D), except HA-RNMT-MTD replaced HA-RNMT. (F) as (D), except RAM 56–118 replaced RAM.

Since RNMT is essential for mammalian cell viability, the mechanism by which RAM regulates RNMT expression was investigated. Although inhibiting RAM expression can regulate expression levels of other transcripts (Figure 6B), it did not alter the RNMT transcript level (Figures 6A and 7A).

The effect of RAM in regulating RNMT had been observed with endogenous proteins (Figures 5A–C). RAM overexpression was also observed to increase RNMT expression following exogenous expression of both genes in 293 cells (Figure 7B). When expressed alone, HA-RNMT expression was relatively low, however when coexpressed with FLAG-RAM, a significant increase in HA-RNMT expression was observed (Figure 7B, left panel). The effect of RAM on RNMT was again independent of transcript level (Figure 7B, right panel). This effect was also independent of cap methyltransferase activity since expression of RAM stablized a methyltransferase dead RNMT mutant (MTD), equivalently to wild-type RNMT (Figure 7C).

We found no evidence that RAM regulated RNMT translation, for example, RAM expression did not alter the migration of RNMT transcripts in polysome profiles (data not shown). Since RAM was observed to regulate endogenous and exogenous RNMT expression, it is likely that RAM protein stabilizes RNMT protein. During the investigation of protein stability, protease inhibitors are often invaluable, however an extensive panel of proteasome and protease inhibitors was found to have no effect on RNMT protein expression, regardless of RAM expression (not shown). It is a possibility that RNMT stability is regulated by several mechanisms.

To strengthen the evidence that RAM stabilizes RNMT, the mechanism was investigated in vitro. RNMT was translated in vitro either alone or while RAM was also being translated (Figure 7D). RNMT accumulation was significantly increased when RAM was translated simultaneously (Figure 7D, left panels). This effect was independent of changes in RNMT transcript level (Figure 7D, right panels), and did not require RNMT to be catalytically active (Figure 7E). Consistent with the hypothesis that RAM regulates RNMT stability, a RAM mutant defective for RNMT binding did not increase RNMT expression (Figure 7F).

## Discussion

Nascent RNA pol II transcripts receive a methyl cap while being transcribed, directing them to the processes required for their expression and maturation and protecting RNA against degradation (Bentley, 2005; Furuichi and Shatkin, 2000; Moore and Proudfoot, 2009; Shatkin and Manley, 2000). Cap methylation is catalyzed by the enzyme RNMT (RNA guanine-7 methyltransferase) in humans and orthologs in other eukaryotes. Here we report that the minimal human cap methyltransferase consists of RNMT and a previously uncharacterized 14 kDa protein, Fam103a1, which we designated as RAM. In cells, the vast majority of RAM and RNMT were found in a complex, and monomeric RNMT and RAM were undetectable. We provide several lines of evidence that RAM is an obligate RNMT activator. In vitro, RAM significantly stimulated recombinant RNMT activity and RAM was required for cellular RNMT activity. In vivo, inhibition of RAM expression resulted in a reduction in the levels of methyl caps found on endogenous cellular transcripts, even in the presence of wild-type levels of RNMT expression, demonstrating that cellular RNMT cannot function without RAM. Consistent with being an activator of cellular cap methylation, RAM was required for maintaining levels of endogenous RNA pol II transcripts and for cell viability.

Human RAM protein consists of 118 amino acids, without regions of homology to previously described functional domains. The RAM N terminus is notable for being relatively well conserved among the RAM orthologs. The RAM N terminus interacts with RNMT and was sufficient to activate RNMT in vitro equivalently to full-length RAM. Since it did not bind to RNA or enhance RNMT binding to RNA, we speculate that the RAM N terminus may alter the conformation of the active site. Structural studies are required to explore this hypothesis.

The C terminus of RAM also does not contain regions homologous to previously described functional domains but is enriched in asparagine, arginine, tyrosine, and glutamine, amino acids previously identified to be enriched in the RNA-binding domain of hnRNPU (Kiledjian and Dreyfuss, 1992). We demonstrated that RAM interacts with RNA via the C terminus. RNMT binds to its substrate, the inverted guanosine cap, weakly, and this affinity increases when the cap is present on a transcript (Wen and Shatkin, 2000). We observed that RAM bound to transcripts as a monomer, via the C terminus. When RAM and RNMT were presented to transcripts as a complex, their affinity for RNA significantly increased.

Following the observation that RAM is an RNA-binding protein, we expected that this domain would stimulate cap methylation by increasing recruitment of the RNA substrate. We were surprised to find that in the in vitro cap methyltransferase assay, the RAM RNA-binding domain was not required to stimulate cap methyltransferase activity. However, we note that this assay is performed on a transcript with one sequence, whereas in vivo there is a huge variety of substrate sequences, that is, the 5′UTRs (5′ untranslated regions), and therefore in vivo it is possible that RAM enhances recruitment of specific transcripts to RNMT.

A 7-methylguanosine cap is found on RNA pol II transcripts in all eukaryotic species investigated, and cap methyltransferase orthologs have been isolated from several species including *ABD1* in *S.cerevisiae* and Pcm1 in *S.pombe.* The conserved nature of these orthologs is reflected in the fact that the human cap methyltransferase RNMT can rescue the viability of Abd1p-deficient *S.cerevisiae* (Saha et al., 1999). We identified orthologs of RAM in vertebrates only; however, these were well conserved. For example, *H.sapiens* RAM and *X.tropicalis* RAM have 87.5% identity. The evolution of a cap methyltransferase activating subunit may have occurred to contend with the expanded gene repertoire or the increasing complexity of 5′UTRs associated with the evolution of vertebrates. It remains possible that in lower organisms, although RAM orthologs have not been identified, proteins with an unrelated amino acid sequence may perform an analogous function.

Another example of a cap methyltransferase activating subunit has been identified previously in poxviruses. A subset of eukaryotic DNA viruses, including the poxviruses, encode their own enzymes for methyl cap formation (Shuman, 2002). Poxviruses encode a RNA polymerase and the three enzymes required for methyl cap formation on a single polypeptide, D1. However the cap methyltransferase has minimal activity without its activating subunit, D12 (Mao and Shuman, 1994). Poxvirus D12 and RAM have little sequence homology; however, both have an isoelectric point above 8.8, and their basic nature may promote RNA binding and activation of the cap methyltransferase.

In summary, RAM/Fam103a1 is an essential component of the gene expression machinery required for mRNA cap methylation.

## Experimental Procedures

### Cell Culture

Cells were cultured in DMEM supplemented with 10% Foetal Bovine Serum, in 5% CO_2_ at 37°C. INI-HA-RNMT and vector control were transduced into cells by retroviral infection according to standard protocols and selected using 0.5 mg/ml G418. HeLa cells with Doxycyline-inducible HA-RNMT were created using the T-REx system (Invitrogen). Transfection reagents and siRNAs from the siGenome range (Dharmacon) that target single sites or nontargeting controls were used. Gene Juice (Novagen) was used to transfect cells with DNA. Cells were counted using a haemocytometer.

### Immunoprecipitation and Western Blotting

Lysis buffer (10 mM Tris [pH 7.05], 50 mM NaCl, 50 mM NaF, 10% glycerol, 0.5% Triton X-100 and protease inhibitors) was used to extract cellular protein. All immunoprecipitations were performed at 4°C. For the large scale RNMT purification, 50 mg of cell extracts were precleared for 2 hr using 50 μg murine IgG-conjugated agarose (Sigma) and subsequently incubated with 50 μg monoclonal anti-HA antibody-conjugated agarose (Sigma) overnight. Immunoprecipitates were washed in lysis buffer and resolved by 4%–12% SDS-PAGE. The gel was fixed (40% methanol, 7% glacial acetic acid) and stained with brilliant blue G-Colloidal (Sigma) for 2 hr. For endogenous protein immunoprecipitation, 0.5 mg cell extracts were incubated with 1 μg sheep polyclonal anti-RNMT, RAM, or GST (control) antibodies for 6 hr. SDS-PAGE was used to resolve 10 μg input and 30% immunoprecipitates. The immunodepletions were performed using three rounds of immunoprecipitation following which 10 μg of cell extract was resolved by SDS-PAGE. Western blots were performed according to standard protocols. Antibodies were raised against full-length recombinant human RNMT, human RAM/Fam103a1, and GST in sheep, and sera were affinity purified on the recombinant protein.

For exogenous protein immunoprecipitation, 1.5 × 10^6^ 293 cells were transfected with 3 μg pEGFP-RAM FL, pCDNA4-HA-RNMT FL, deletion mutants, or relevant empty vector using calcium phosphate. Forty-eight hours posttransfection, 0.5 mg cell extract was subjected to immunoprecipitation for 6 hr using 1 μg of mouse anti-HA antibody-conjugated agarose (Sigma) or 1 μg anti-GFP antibody (Roche) plus 25 μl Protein A/G Sepharose. SDS-PAGE (12%) was used to resolve 20 μg inputs and 30% immunoprecipitates.

In order to investigate the effect of RAM expression on RNMT, 8 μg pcDNA3.1 FLAG-RAM (Fg-RAM), and/or 2 μg pcDNA3.1 HA-RNMT/HA-RNMT MTD (methyltransferase-dead; V201A, G207A, D211A), and/or relevant vector controls were transfected into 1.5 × 10^6^ 293 cells. Forty-eight hours later cells were lysed and western blots performed on 10 μg cell extract or on anti-RAM antibody immunoprecipitates from 2 mg cell extracts.

### Mass Spectrometry

Coomassie stained gel bands were excised, washed, and incubated overnight with trypsin. Peptides were extracted and separated on a nanoLC system, which was attached to a mass spectrometer with a nano flow ionization source attached. The identification of the following peptides was performed by MS/MS Ion Search: FEEMFASR, FTENDKEYQEYLK, RPPESPPIVEEWNSR, and WGWPSDNR.

### Gel Filtration

HeLa cells were resuspended in 20 mM HEPES (pH 7.5), 1.5 mM MgCl_2_, 10 mM KCl, passed through a 20G needle 20 times, subjected to three freeze-thaw cycles in a dry ice/isopropanol bath, and centrifuged at 14,000 g for 10 min at 4°C. The supernatant was centrifuged at 100,000 g for a further 60 min at 4°C. 1 mg cell extract was resolved on a Superdex s200 10/30 HR column (Pharmacia) in 50 mM Tris–HCl (pH 8), 6 mM KCl, and 1.25 mM MgCl_2,_ using an AKTA FPLC (Pharmacia).

### Recombinant Protein Production

pGEX-6P1-based vectors were transduced into BL21(DE3) *E.coli*. When a 1 l culture A600 was 0.6, expression of recombinant protein was induced with 0.5 mM IPTG (isopropyl-β-D-thiogalactopyranoside) at 25°C for 16 hr. Cells were harvested by centrifugation, resuspended in 15 ml lysis buffer (50 mM Tris [pH 7.5], 150 mM NaCl, 1% Triton X-100, 1 mM EDTA, 1 mM EGTA, 0.1% beta-mercaptoethanol, 0.2 mM PMSF, 1 mM benzamidine, 100 μg/ml leupeptin, 1 mg/ml lysozyme) and sonicated on ice for 30 s, 6 times. Insoluble material was removed by centrifugation for 20 min at 40,000 g. 1.5 ml glutathione-sepharose resin was incubated with the soluble material for 1 hr, washed in wash buffer (50 mM Tris [pH 7.5], 150 mM NaCl, 0.01% Triton X-100, 0.1% beta-mercaptoethanol, 0.2 mM PMSF, 1 mM benzamidine, 100 μg/ml Leupeptin) and protein eluted in 5 ml of 50 mM glutathione wash buffer. Resin was incubated overnight in wash buffer containing 200 μg of prescission protease and dialyzed. Recombinant proteins were resolved by SDS-PAGE (Figure S4).

### Recombinant Protein Interaction

10 μg of RAM-GST and GST bound to glutathione sepharose was rotated at 4°C with 1 μg RNMT in lysis buffer for 2 hr. Glutathione sepharose was recovered by centrifugation and washed 6 times in lysis buffer. Proteins were eluted using Laemmli buffer and analyzed by western blot.

### Immunofluorescence

All incubations were performed in 0.2%BSA/PBS at room temperature unless stated. Cells were fixed in 4% paraformaldehyde for 10 min, blocked with 10% donkey serum for 20 min, and incubated in 20 μg/ml polyclonal sheep anti-RAM or RNMT antibodies for 1 hr, then washed and incubated with 4 μg/ml Alexa Fluor 488-conjugated Donkey Anti-Sheep antibodies for 45 min. Cells were counterstained 1 μg/ml DAPI (4′,6-diamidino-2-phenylindole), mounted in 2.5% DABCO, and visualized by fluorescence microscopy (Zeiss LSM 700).

### RNA Band Shift Assay

55 nt transcripts were in vitro transcribed using 20 U T7 RNA polymerase (Promega) according to the manufacturer's instructions from 500 ng EcoRI-linearized pGEM-CEM4 and labeled with [α-32P]-GTP (3000 Ci/mmol; Hartmann) and 1 mM cap analog (NEB). 1/50 purified transcripts were incubated for 1 hr at 4°C with 0.5–2 pmol recombinant RAM and/or 2 pmol RNMT in 20 mM Tris (pH 7.5), 50 mM KCl, 10 mM MgCl_2_, 0.1% Triton X-100, 5 mM DTT, 1 mg/ml BSA, 7.5% glycerol, 20 U RNasin (Promega) and 50 μM s-adenosyl homocysteine. Complexes were resolved at 4°C by native PAGE (4.5% acrylamide, 25 mM Tris [pH 8.3], 190 mM glycine, 1 mM EDTA, and 0.1% Triton X-100) and visualized by phosphoimaging (Molecular Dynamics). When relevant, 700 ng affinity-purified, anti-RAM, RNMT, and GST sheep antibodies were incubated with complexes 15 min prior to PAGE resolution.

### Cap Methylation Assay

Cap methylation assays were performed according to Cowling (2010). Briefly, an in vitro transcribed 55 nt transcript, capped with α^32^P-GTP was the substrate for the methylation reaction. For assays using recombinant protein, 20 nM RNMT or 5–80 nM RAM were used. Proteins were incubated for 5 min at room temperature prior to addition of 10 ng RNA substrate and 100 nM S-adenosyl methionine and further incubated at 37°C for 10 min or the time indicated. For assays using cellular proteins, 2 μg cell extract or HA-RNMT immunoprecipitated using 5 μl anti-HA agarose (Sigma) from 0.5 mg 293 cell line extract were used (Figure 1A). When relevant, affinity-purified, polyclonal anti-RAM and GST antibodies were added to the reaction for 5 min prior to addition of RNA substrate. Following the reaction, RNA was purified by phenol:chloroform extraction, acetate precipitated, and resuspended in 4 μl of 50 mM NaAcetate (pH 5.5) and 0.25 U P1 nuclease (Sigma) for 30 min at 37°C to release free GpppG and m7GpppG. Cap structures were resolved by 0.4 M ammonium sulfate on PEI cellulose plates. Labeled GpppG and m7GpppG spots were visualized and quantified by autoradiography. Standards were visualized by UV light to establish correct migration.

### Polysome Profiling

HeLa cells were incubated in 100 mg/ml cycloheximide for 3 min and extracts prepared by dounce homogenization (Wheaton B) in polysome extraction buffer (10 mM Tris [pH7.5], 15 mM MgCl_2_, 0.3 M NaCl, 1% Triton X-100, 100 mg/ml cycloheximide, 100 U/ml RNasin). Extracts were normalized by OD 260 nm and layered onto 11 ml of 10%–50% sucrose steps and centrifuged at 30K rpm for 2 hr at 4°C. The sucrose steps were fractionated into twelve 1 ml fractions, and OD 254 nm was monitored.

### In Vivo Methyl Cap Immunoprecipitation

dT-purified RNA was immunoprecipitated with 10 μl anti-7-methyl guanosine (Cole and Cowling, 2009). Transcripts were analyzed by real-time PCR and immunoprecipitates expressed relative to inputs.

### In Vitro Translation

In total 1μg of pcDNA3.1 RNMT or RNMT MTD (V201A, G207A, D211A), and/or pcDNA3.1 RAM or RAM C (amino acids 56–118) and relevant controls, were in vitro translated in 10 μl TNT coupled reticulocyte lysate system (Promega) and labeled with [^35^S]methionine. Protein was resolved by SDS-PAGE and quantified by phosphoimaging. RNMT translated relative to amount of template was calculated. RNA was extracted from the same reactions and used as a substrate for RT-PCR using primers specific for HA-RNMT.

### Real-Time Polymerase Chain Reaction

For RT-PCR performed on in vitro transcriptions, transcript levels are expressed relative to the amount of DNA template introduced. Real-time PCR was performed using Quanta Biosciences SYBR Green FastMix for iQ. Primers used are available on request. PCR products were sequence verified.

### Cloning

Full-length and deletion mutants of *H.sapiens* RNMT were subcloned from INI-HA-RNMT (Cowling, 2010) into pCDNA4 by PCR, including an HA tag at the N terminus, and further subcloned. Fam103a1/RAM was cloned by PCR from HeLa cell cDNA into pEGFP-N3 (C-terminal GFP), and pcDNA3.1 with an N-terminal FLAG (Fg) tag. RNMT and RAM proteins and mutants were cloned into pGEX 6P-1 (N-terminal GST). All constructs were sequence verified.

## Supplemental Information

Document S1. Five Figures
