# Supplementary material for: RAM/Fam103a1 Is Required for mRNA Cap Methylation
Source: Mol Cell. 2011 Nov 18;44(4):585–96. doi: 10.1016/j.molcel.2011.08.041 (PMC3235549; doi:10.1016/j.molcel.2011.08.041)
Supplement: Document S1. Five Figures [file mmc1.pdf]

Molecular Cell, Volume *44*

Supplemental Information

RAM/Fam103a1 Is Required for mRNA Cap Methylation

Thomas Gonatopoulos-Pournatzis, Sianadh Dunn, Rebecca Bounds, and Victoria H. Cowling

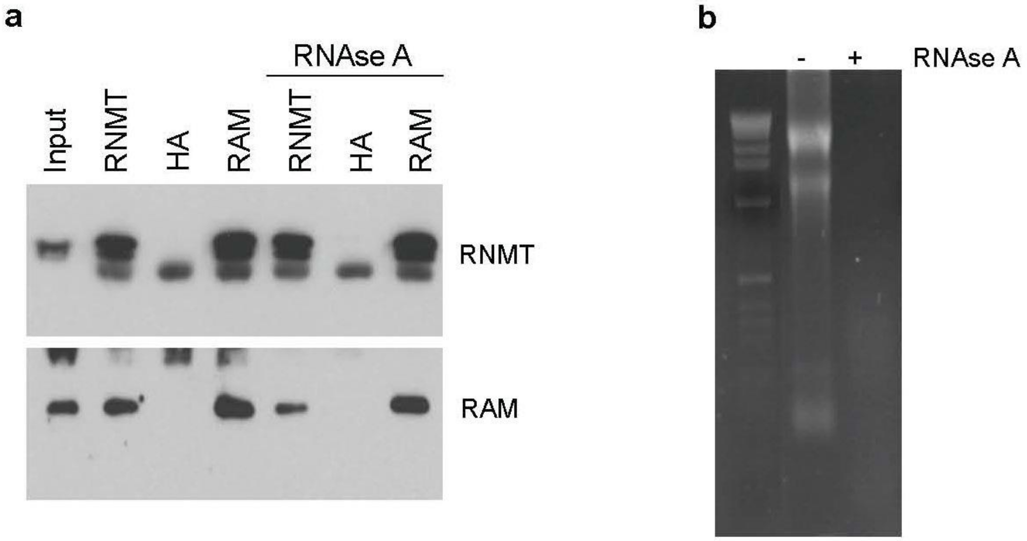

Figure S1, Related to Figure 1. RNAse Treatment Does Not Inhibit the RNMT-RAM Interaction

(a) HeLa cell extracts were subject to immunoprecipitation using polyclonal anti-RNMT, RAM and HA (negative control) antibodies, as indicated above the panel. Immunoprecipitations were performed as described in experimental procedures followed by three washes in RNAse A buffer. 10μg of RNAse or control were incubated with immunoprecipitates for 60 mins at 4°C, as indicated. RNMT and RAM were detected in the immunoprecipitates by western blot. Input lane is 20μg cell extract.

(b) The activity of RNAse A was confirmed by incubating 1ug cellular RNA with (+) or without (-) 10μg RNAse A under the same conditions. RNA was purified and resolved by gel electrophoresis.

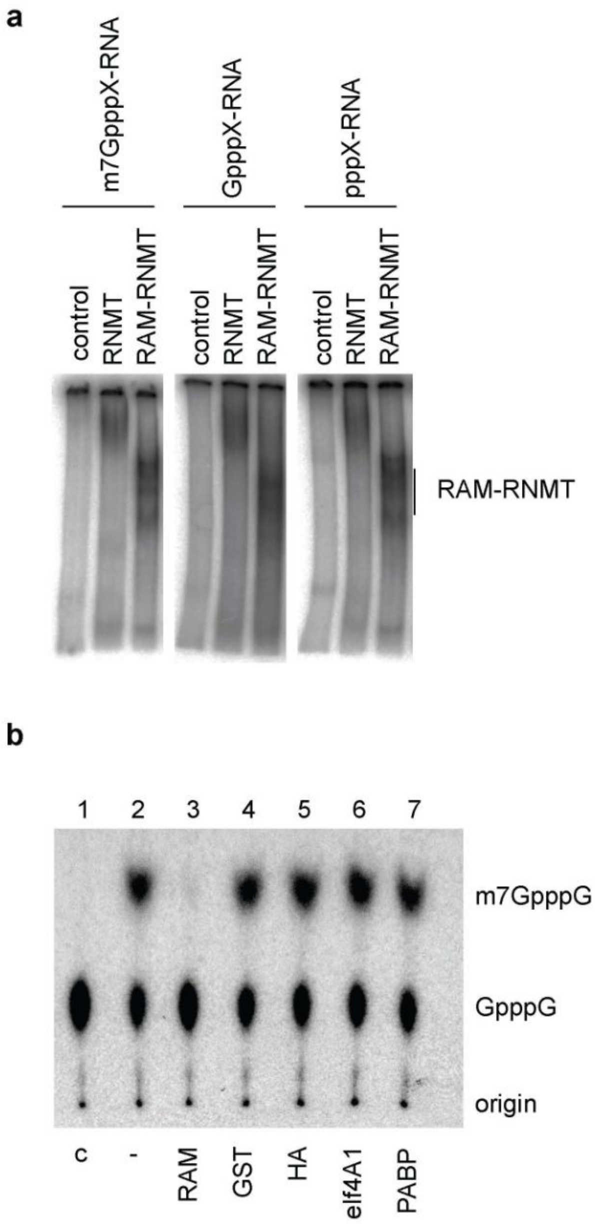

Figure S2, Related to Figure 3. RAM-RNMT Complexes Bind Equivalently to Uncapped, Capped, and Methyl Capped RNA

(a) RNA bandshift assay was performed as described in the experimental procedures except the RNA probe was methylcapped (m7GpppX), capped (GpppX), or uncapped (pppX).

(b) Anti-RAM antibodies inhibit the cap methyltransferase activity of cell extracts. Cap methyltransferase assay was performed on 1μg 293 cell extract (lanes 2-7), as in fig. 4.g. Prior to the assay, extracts were incubated with antibodies raised against RAM, GST, HA, eIF4A1, PABP, as indicated.

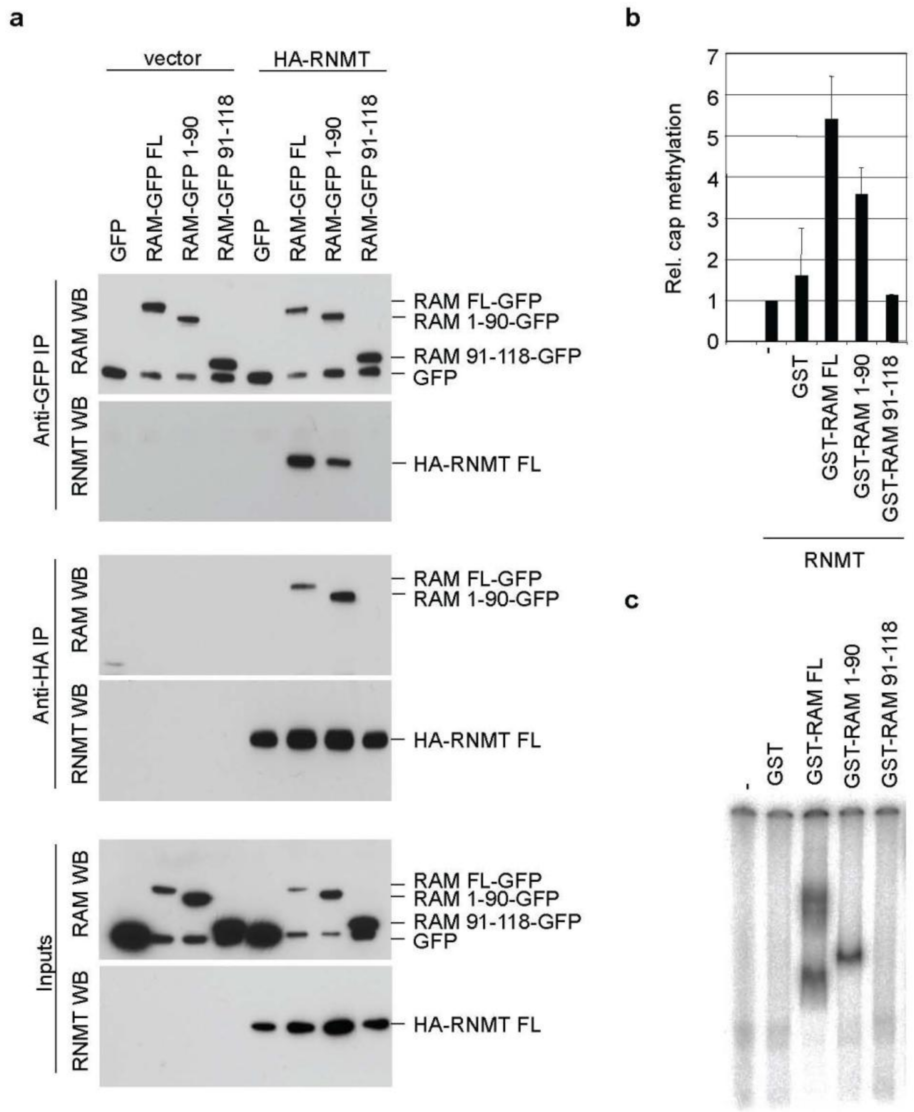

Figure S3, Related to Figure 4. RAM 1-90 Interacts with RNMT and RNA, and Activates Cap Methylation

(a) 293 cells were transfected with the combinations of pEGFP-RAM and pCDNA-HA-RNMT, deletion mutants and vector controls, as indicated. Immunoprecipitations were performed with anti-HA and anti-GFP antibodies. Western Blots were performed to detect RAM and RNMT in inputs (lower panels), anti-HA antibody immunoprecipitates (middle panels), and anti-GFP antibody immunoprecipitates (upper panels).

(b) *In vitro* cap methyltransferase assay performed as described in experimental procedures using 20nM recombinant proteins indicated.

(c) RNA band shift assay performed as described in experimental procedures using 2pMoles recombinant protein indicated.

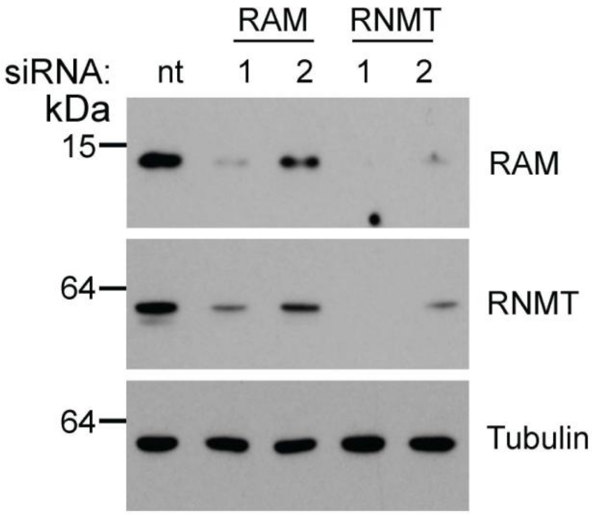

Figure S4, Related to Figure 5. RNMT-Directed siRNA Inhibits RAM Expression

HeLa cells were transfected with 2 independent siRNAs directed against RAM and RNMT, or non-targeting control (nt). 48hrs following transfection, RAM RNMT and b-Tubulin were detected by Western Blot.

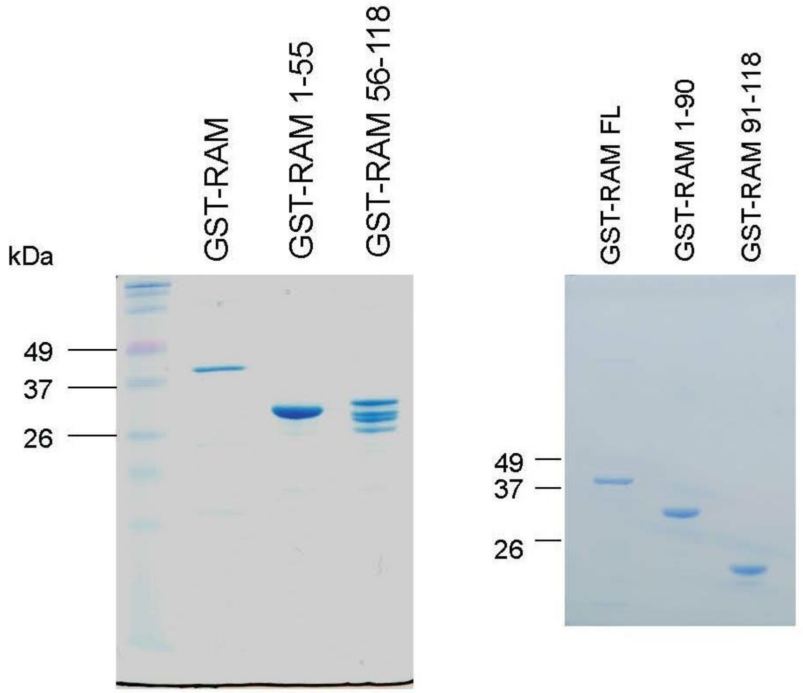

Figure S5, Related to Experimental Procedures. Recombinant GST-RAM and Mutants Resolved by SDS-PAGE and Stained with Coommassie Blue
